# Hydrostatic Pressure Enhances Chondrogenic Differentiation of Mesenchymal Stem Cells in Silk Fibroin-Based 3D Bioprinted Hydrogels

**DOI:** 10.1021/acs.biomac.5c00048

**Published:** 2025-05-20

**Authors:** Jennifer Fritz, Anna-Christina Moser, Alexander Otahal, Karina Kramer, Salih Casurovic, Andreas H. Teuschl-Woller, Stefan Nehrer

**Affiliations:** † Center for Regenerative Medicine, 31227 University for Continuing Education Krems, Dr.-Karl-Dorrek-Straße 30, Krems 3500, Austria; ‡ 125042 Austrian Cluster for Tissue Regeneration, Donaueschingenstrasse 13, Vienna 1200, Austria; § Department of Life Science Engineering, University of Applied Sciences Technikum Wien, Höchstädtplatz 6, Vienna 1200, Austria; ∥ Department of Orthopaedics and Traumatology, Universitätsklinikum Krems, Mitterweg 10, Krems 3500, Austria

## Abstract

The human meniscus experiences mechanical forces and converts axial loads into hoop stresses. Meniscus injuries and meniscectomies can compromise this function, and therefore, meniscus implants are required. To assess their performance *in vitro*, it is crucial to recreate a physiological environment. Therefore, we investigated the effect of TGFβ-3-supplemented and TGFβ-free cyclic hydrostatic pressure (HP) up to 10 MPa on 3D bioprinted silk fibroin (SF) polymer-based hydrogels. The bioink was seeded with human infrapatellar fat pad-derived MSCs and supplemented with an extracellular matrix and gelatin. We found that HP stimulation did not alter cell-free biomaterial maturation, while it partially stimulated metabolic activity and cell proliferation. Remarkably, TGFβ-3-supplemented HP led to the highest expression levels of chondrogenic markers, followed by TGFβ-3-supplemented unloaded incubation and then TGFβ-free HP. Despite the low cell density, the combined exposure to TGFβ-3 and HP also facilitated localized glycosaminoglycan and collagen deposition, demonstrating promising prospects for future meniscus regeneration.

## Graphical abstract

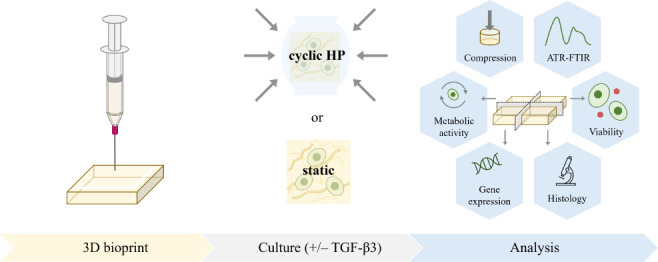

## 1 Introduction

Traumatic and degenerative meniscus injuries are among the most common knee pathologies.^1,2^ The consequences of an injured meniscus can include pain, disability, cartilage loss, and the risk of developing osteoarthritis (OA).^1,3^ Management options range from conservative treatment to surgical interventions such as meniscectomy, meniscal repair, and allograft implantation.^2,4^ The limitations of current treatment options create an urgent need for alternative therapeutic strategies. 3D bioprinted meniscal tissue provides a personalized and promising alternative, as it could replicate the intricate meniscus architecture and promote natural regeneration. Previous attempts to formulate bioinks for 3D bioprinting of meniscus tissue have already investigated collagen, decellularized extracellular matrix (ECM), gelatin and gelatin methacrylate, alginate, cellulose, and hyaluronic acid as matrices for cell encapsulation.^5–9^ In addition, a variety of polymers, including SF and SF methacrylate, have been explored for cell-free 3D printing of meniscus tissue.^10,11^ Several of these studies have demonstrated promising outcomes in terms of mechanical strength, cell viability, and tissue formation. However, the conventional method of statically incubating the 3D bioprinted constructs does not accurately mimic the dynamic and complex conditions found within the human knee joint.

In the course of daily activities, the knee joint is subjected to forces that can reach up to five times the body’s weight and encompass different types, such as shear, tension, compression, and HP.^5^ These forces directly influence the biological response of meniscal cells and tissue organization.^12^ Therefore, the appropriate mechanical loading through tension, compression, HP, perfusion, or a combination of these methods could significantly impact the development and remodeling of the tissue.^13,14^ In general, the meniscus experiences 3–10 MPa of HP with an adult walking cadence of ≥100 steps/min (∼1.7 Hz).^15,16^ During joint loading, the high water content and low permeability of the meniscus allow fluid-phase pressurization, whereby HP exerts uniform compression on all surfaces of cells and tissues.^13,17^ By simulating these physiological conditions during the incubation of a meniscus scaffold, valuable insights into the scaffold’s potential for clinical application could be gained. One approach to mechanical stimulation involves high-HP bioreactors, allowing the constant or dynamic application of HP by pressurization with a piston, a compression fluid, or the cultivation medium.^18^

HP has already been investigated for its effects on human MSCs and meniscus scaffolds. Studies on aggregates or pellets of human MSCs have shown that the application of HP increases glycosaminoglycan (GAG) and collagen production, as well as sex-determining region Y (SRY)-box 9 (*SOX9*), aggrecan (*ACAN*), and collagen type 2 (*COL2A1*) expression.^19,20^ Similar effects of HP on the upregulation of genes for chondrogenic differentiation were also observed in studies where human MSCs were embedded in gellan gum hydrogels and collagen scaffolds.^21–24^ The specific application of cyclic HP to create meniscus tissue with human meniscal cells was reported to increase collagen and GAG deposition in poly-L-lactic acid constructs and to increase collagen type 1 (*COL1A1*) expression in alginate beads.^25,26^ Interestingly, the mechanical properties of the poly-L-lactic acid constructs could also be enhanced by the combined application of HP and growth factors.^25^ These studies highlight the important role of physiological HP not only in chondrogenic differentiation but also in biomaterial maturation.

Therefore, we used a bioreactor with an oil reservoir for compression to apply HP in cycles of 0.5–10 MPa to 3D bioprinted hydrogels. The SF-based hydrogels containing infrapatellar fat pad-derived mesenchymal stem cells (IFP-MSCs), decellularized bovine meniscus ECM and porcine gelatin had already been optimized in our previous study.^27^ To the best of our knowledge, no previous study has focused on cyclic hydrostatic stimulation of SF-based 3D bioprinted hydrogels. Our investigation included a comparative analysis of the impact of HP on biomaterial maturation in 3D-printed cell-free controls and on the proliferation and differentiation of IFP-MSCs within 3D bioprinted constructs, relative to static incubation. We hypothesized that physiologic HP could enhance meniscus tissue formation by promoting chondrogenic differentiation as well as ECM deposition in SF-based 3D bioprinted hydrogels, thereby improving their potential for meniscus regeneration.

## 2 Material and Methods

### 2.1 Preparation of SF and ECM

Bombyx mori silk cocoons (Seidentraum, Germany) were processed into SF solution following published protocols.^28^ Concisely, 5 g of cocoons was cut and degummed in 2 L of boiling 0.02 M Na_2_CO_3_ (Sigma-Aldrich, Austria) for 1 h. The fibers were spread out after washing in ultrapure water (UPW) and dried at room temperature (RT) for 48 h. Then, 20% (w/v) were dissolved in 9.3 M LiBr (Sigma-Aldrich, Austria) for 3 h at 60 °C. The solution was dialyzed against UPW for 3 days in a Spectra/Por dialysis membrane (Carl Roth, Germany). The SF concentration was increased by storing the membrane with the SF solution at 4 °C for at least 1 week. The solution was centrifuged for 10 min, and the supernatant concentration was determined by drying for 12 h at 90 °C. The mean of three samples gave the final SF concentration. The SF solution was stored at 4 °C until further use.

Bovine menisci were procured from a slaughterhouse and subjected to decellularization and digestion according to adapted published procedures.^29,30^ Briefly, cut menisci were lyophilized for 3 days and milled. 1 g was stirred in 40 mL of 1X PBS (Thermo Fisher Scientific, Austria) with 1% (v/v) Triton X-100 (Sigma-Aldrich, Austria) at 4 °C for 3 days. The tissue was washed in 1X PBS and transferred to 40 mL of an RNA-DNA enzyme extraction buffer. The buffer contained 2.5 kU benzonase (Sigma-Aldrich, Austria) and 8 mM MgCl_2_ (Sigma-Aldrich, Austria) in UPW, adjusted to pH 8. The RNA and DNA removal was carried out at 37 °C for 24 h prior to the final six washes in 1X PBS. Then, the tissue was lyophilized and milled again. 5% (w/v) ECM powder was digested in 0.1 M HCl with 0.5 mg/mL porcine pepsin (Sigma-Aldrich, Austria) for 2 h at 37 °C. The neutralized solution was lyophilized and stored at 4 °C until further use. The effectiveness of decellularization and the maintenance of the collagen content have already been demonstrated in our previous study.^27^

### 2.2 IFP-MSC Preparation

IFP-MSCs were obtained from three male patients undergoing knee surgery with written informed consent and ethical approval (GS4-EK-4/763-2021). The donor characteristics are provided in Table 1. The youngest patient’s diagnosis included a rupture of the anterior cruciate ligament (ACL) and lateral and medial menisci. The middle-aged patient suffered from osteochondritis dissecans (OCD), which describes the detachment of a piece of the subchondral bone and articular cartilage and can progress to osteoarthritis (OA.^31^ The oldest patient had OA. Cell isolation was performed according to published protocols.^32,33^ 10 g of minced IFP tissue was digested with 9000 units of collagenase I (Sigma-Aldrich, Austria) in Gibco DMEM (Thermo Fisher Scientific, Austria) for 2 h at 37 °C. The suspension was then filtered through a cell strainer (40 μm, Thermo Fisher Scientific, Austria), and the cells were washed in 1X PBS and resuspended in MSC growth medium (Gibco DMEM with high glucose, GlutaMAX, and pyruvate (Thermo Fisher Scientific, Austria), 10% (v/v) FCS, 1% (v/v) nonessential amino acids (both from Thermo Fisher Scientific, Austria), 2% (v/v) Penicillin/Streptomycin, 1% (v/v) Amphotericin B, and 1 ng/mL Fibroblast Growth Factor-Basic (all from Sigma-Aldrich, Austria)). Cells were expanded at 37 °C with 5% CO_2_, detached with accutase, centrifuged, and diluted in freezing media (10% (v/v) DMSO (Sigma-Aldrich, Austria) and 90% (v/v) FCS). 10^6^ cells in 1 mL of freezing media were stored in a liquid nitrogen tank. Cells from passage 3 to 5 were thawed for the experiments (Table 2). For thawing the cells, the samples were transferred to a 37 °C water bath for 2 min. MSC growth medium was added to the thawed samples before centrifugation. The cells were then seeded in MSC growth medium at 37 °C with 5% CO_2_ to be expanded over two passages. Cells from passage 5 to passage 7 were seeded into the bioink for 3D bioprinting.

**Table 1:** IFP-MSC Donor Characteristics

Donor	Sex	Age [y]	Weight [kg]	Height [cm]	Disease	Duration of disease
1	m	22	80	175	Ruptured ACL, lateral and medial meniscus	>10 months
2	m	42	80	184	OCD	>10 years
3	m	82	80	178	OA	>5 years

**Table 2:** IFP-MSC Growth Characteristics^a^,^b^

Donor	Thawed passage	Passage seeded in bioink	Population doublings between passages	Mean doubling time [d]
1	3	5	4	6
2	4	6	5	4
3	5	7	5	4

a IFP-MSCs from three donors were expanded in 2D culture over two passages before they were used for 3D bioprinting.

b The population doublings and mean doubling times over these passages were calculated.

### 2.3 3D-Bioprinting

A SF10-ECM5-G3 solution containing 10^6^ IFP-MSCs/mL and 10 U/mL HRP was aspirated into a syringe, which was then transferred to the 3D bioprinter. The solution was allowed to gel for 30 min at 20 °C before cuboids with a height of 4 mm, side lengths of 20 mm, and a grid spacing of 0.5 mm were extruded into a 6-well plate. The printing process utilized a 27G-sized nozzle operating at a speed of 6 mm/s and approximately 30 PSI. Three mL of MSC growth medium with 0.029% (v/v) H_2_O_2_ were added to each well to provide 0.01% (v/v) H_2_O_2_ to each cuboid. The cuboids were incubated at 20 °C for 2 h before the medium was refreshed and the plate was transferred to 37 °C. One sample was subjected to all tests on day 0, while the other samples were incubated until day 14 or day 28. Starting from day 1, half of the samples were exposed to HP stimulation in a bioreactor, while the other half were continuously incubated statically at 37 °C with 5% CO_2_. Each incubation condition included samples immersed in MSC growth medium and samples immersed in chondrogenic differentiation medium (Gibco DMEM with high glucose, GlutaMAX, and pyruvate (Thermo Fisher Scientific, Austria), 1% (v/v) ITS, 100 nM dexamethasone, 50 μg/mL ascorbic acid, 2% (v/v) Penicillin/Streptomycin, 1% (v/v) Amphotericin B (all from Sigma-Aldrich, Austria), 10% (v/v) FCS, 1% (v/v) nonessential amino acids (both from Thermo Fisher Scientific, Austria), and 5 ng/mL TGFβ-3 (PeproTech, USA)). The medium was changed twice a week under all conditions. The experiment was performed three times with IFP-MSCs from three donors, with the initial trial including a negative cell-free control.

### 2.4 HP Stimulation in Bioreactor

The bioreactor samples were placed into histology cassettes (Leica, United States), and each cassette was sealed in a bag with 10 mL of MSC growth medium or chondrogenic differentiation medium. The sealing process was repeated until each sample was sealed in four bags. The samples were placed into the prewarmed bioreactor and stimulated with hydrostatic pressure cycles of 0.5–10 MPa at approximately 0.7 Hz for 1 h on 5 days a week. After each stimulation, the bags were taken out of the bioreactor, and the first layer was removed to get rid of the oil. The second layer was removed at the sterile working bench. The bags with two layers remaining were incubated at 37 °C with 5% CO_2_ until the next bioreactor stimulation.

### 2.5 ATR-FTIR Spectroscopy

The negative cell-free controls of the static and HP incubation were subjected to attenuated total reflection-Fourier transform infrared (ATR-FTIR) spectroscopy. The hydrogels were immersed in D_2_O (Carl Roth, Germany) three times for 30 min, before they were placed on the ATR-FTIR device (PerkinElmer, Austria). Four scans with a resolution of 1 cm^–1^ between 450 and 4000 cm^–1^ were averaged. Since the region of interest for SF secondary structures ranges from a wavenumber of 1600 cm^–1^ to 1700 cm^–1^ (amide I region), this region was examined closely, and its baselines were subtracted.

### 2.6 Unconfined Compression

The negative cell-free controls were placed onto the holder of the MFT-5000 multifunctional tribometer (Rtec Instruments, United States). The stamp was lowered until it touched the sample. The samples were subjected to a linearly increasing force of 1 N within 2 min. The applied stress *F*_*z*_ [N] was converted to the stress σ [MPa] with the surface area of the sample *a* [m^2^]. The axial strain *ε* [-] was calculated based on the height change. Subsequently, the Young’s modulus [MPa] was calculated by dividing the stress σ [MPa] by the axial strain *ε* [-]. The mean of the last 10 values of the Young’s modulus before an axial strain of 10% was used to specify one Young’s modulus value.

### 2.7 Metabolic Activity

Each cuboid with IFP-MSCs was quartered to be subjected to metabolic activity measurement, viability assay, gene expression analysis, and histology. One quarter was minced, weighed, and investigated via the XTT assay (Roche Diagnostics, Germany) according to the manufacturer’s protocol. The samples were agitated for 5 h in 250 μL of the assay reagents and 250 μL of MSC growth medium at 37 °C. Then, the supernatant was discarded, and the colored gel pieces were agitated for 1 h in DMSO at 37 °C. The supernatant was used to measure the absorbance at 492 and 690 nm.

### 2.8 Viability Assay with Confocal Microscopy

Vertical hydrogel slices of one-fourth of the cuboids with IFP-MSCs were washed in 1X PBS three times. Then, they were incubated in 500 μL of 1X PBS with 4 μM calcein AM (Thermo Fisher Scientific, Austria) and 8 μM ethidium homodimer-1 (EthD-1) (Thermo Fisher Scientific, Austria) for 1 h at 37 °C. The gels were washed again and visualized with the 10X objective of the TCS-SP multiphoton confocal microscope (Leica, United States).

### 2.9 RNA Extraction and DNA Digestion

One quarter of the cuboids with IFP-MSCs was minced and stored in a MagNA Lyser tube (Roche Diagnostics GmbH, Germany) with 500 μL of TRIzol Reagent (Thermo Fisher Scientific, Austria) in liquid nitrogen. After thawing, 100 μL of chloroform (Thermo Fisher Scientific, Austria) was added to each sample, which was then transferred to the MagNA Lyser (Roche Diagnostics GmbH, Germany). The samples were subjected to one run at 6500 rpm for 20 s. The TRIzol RNA extraction was performed as described in the manufacturer’s instructions. DNA digestion was performed with DNase I (Thermo Fisher Scientific, Austria) according to the manufacturer’s manual. The RNA was stored at – 80 °C until it was used for cDNA synthesis and quantitative polymerase chain reaction (qPCR).

### 2.10 cDNA Synthesis and qRT-PCR

RNA from bacteriophage MS2 (Sigma-Aldrich, Austria) was used for RNA stabilization during cDNA synthesis with the Transcriptor First Strand cDNA Synthesis Kit (Roche Diagnostics GmbH, Germany) according to the manufacturer’s instructions. qPCR was performed using the FastStart Essential DNA Probes Master Kit (Roche Diagnostics GmbH, Germany) following the manufacturer’s guidelines. 1 μL of cDNA, FastStart Probe Master 2x, hydrolysis probe (final concentration 250 nM), and primers (final concentration 900 nM) were applied for qPCR amplification on a Roche LightCycler 96. *COL1A1*, *COL2A1*, collagen type 10 (*COL10A1*), *SOX9*, matrix metalloproteinase-3 (*MMP3*), matrix metalloproteinase-13 (*MMP13*), and *ACAN* were analyzed using glyceraldehyde-3-phosphate dehydrogenase (*GAPDH*) as the reference gene. The Ct (cycle threshold) values were used to calculate the fold change in gene expression in comparison to samples in the MSC growth medium on day 0 by the 2^–ΔΔCt^ method. Primers for the genes were used as previously published.^33,34^

### 2.11 Histology

One quarter of each cuboid with IFP-MSCs from donor 1 was embedded in cryosectioning medium, Tissue-Tek O.C.T. Compound (Sakura Finetek, The Netherlands), using disposable specimen molds (Sakura Finetek, The Netherlands). One OA human meniscus sample was used for comparison. It was obtained from the University Hospital Krems after written informed consent from a patient who underwent total knee arthroplasty (TKA). Ethical approval for the use of human meniscus samples was granted by the Ethics Committee of Lower Austria (GS4-EK-4/763–2021). The meniscus sample and the hydrogel samples were transferred to – 80 °C for at least 24 h. Subsequently, they were sectioned on a Cryostar NX70 cryostat (Thermo Fisher Scientific, Austria) into 10 μm thin slices and placed on adhesive glass slides (Thermo Fisher Scientific, Austria). Three slides per condition were prepared for Hematoxylin–Eosin staining, Alcian Blue staining, and Azan staining. The Hematoxylin–Eosin Fast Staining Kit (Carl Roth, Germany) was used according to the manufacturer’s instructions to stain cell nuclei. Alcian Blue solution (Sigma-Aldrich, Austria) and Nuclear Fast Red (VWR, United States) were applied to stain sulfated glycosaminoglycans (sGAGs) and nuclei by incubating the slides in Alcian Blue for 30 min and in Nuclear Fast Red for 5 min. Collagen was visualized by Azan staining, where Azokarmin (Sigma-Aldrich, Austria) was applied for 15 min, Anilin Free Base (Sigma-Aldrich, Austria) in ethanol for 45 min, phosphotungstic acid for 20 min, and Anilin Blue-Orange G (Sigma-Aldrich, Austria) for 15 min. All stained slides were dehydrated using an ethanol and xylene series before being covered with Eukitt (Sigma-Aldrich, Austria). Pictures were taken with a DM-1000 light microscope (Leica, United States).

### 2.12 Statistical Analysis

The statistical calculations were performed using GraphPad Prism version 9.3.1 (Inc., San Diego, USA). Data were presented as mean ± standard deviation (SD) and tested for normal distribution by the Shapiro-Wilk test. If the data were normally distributed, two-way ANOVA with Tukey’s multiple comparisons test was applied. Significance was attributed when *p* < 0.05 (*), *p* < 0.01 (**), *p* < 0.001 (***), and *p* < 0.0001 (****). If the data were not normally distributed, Kruskal–Wallis tests were performed to compare the different conditions with each other on days 14 and 28, as well as the different time points (days 0, 14, and 28) within one condition.

## 3 Results

### 3.1 Biomaterial Maturation Remains Unchanged by HP

To observe potential differences in the secondary structure of cell-free controls in static and HP incubations, the gels were analyzed by ATR-FTIR spectroscopy. A closer look at the amide I region revealed similar β-sheet peaks under both conditions on days 14 and 28, while no β-sheet peak was detected on day 0 (Figure 1A,B). The application of HP partly led to more pronounced random coil (RC) shoulders compared to static incubation. However, the mean curves still resembled those of static incubation, indicating no significant difference in the secondary structures.

**Figure 1:**
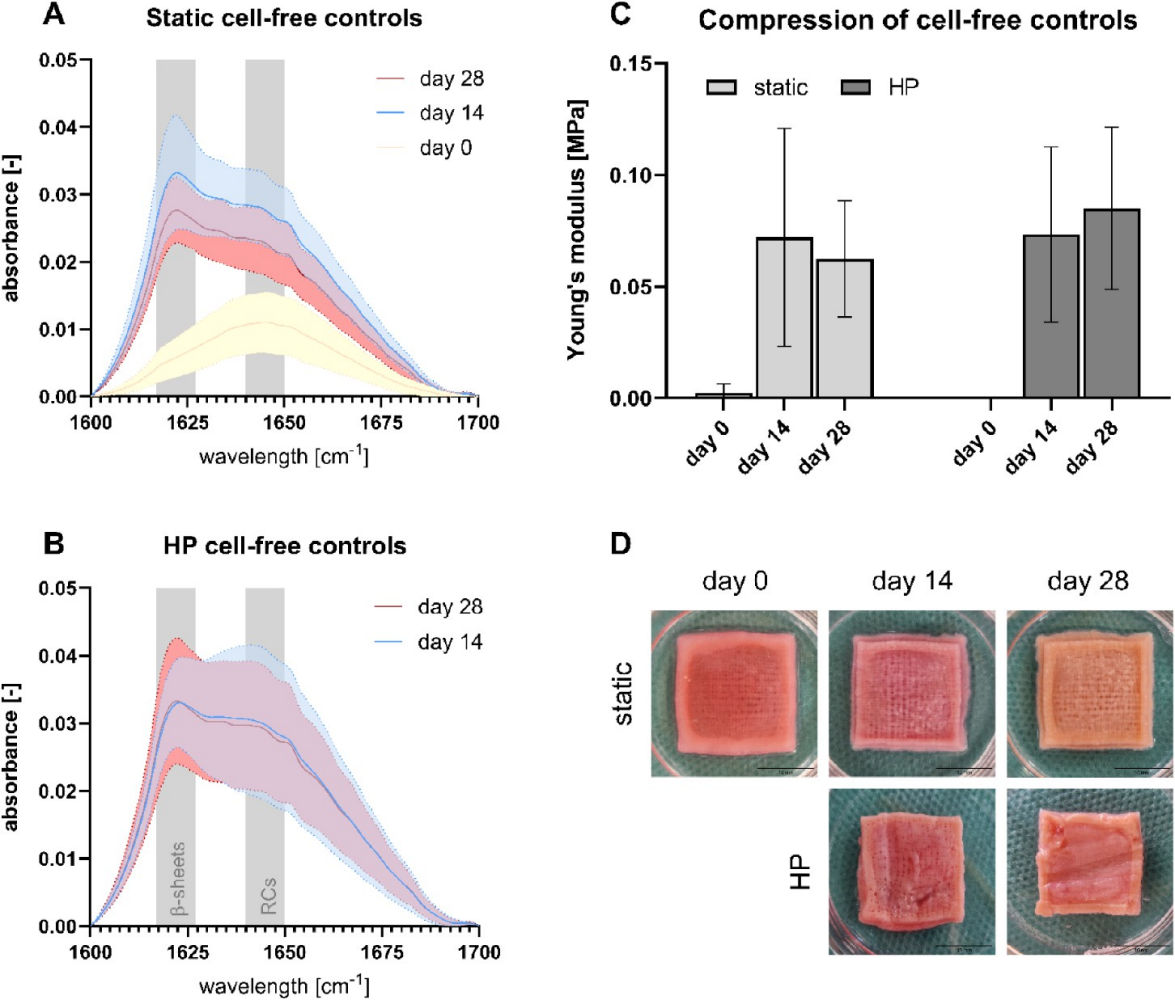
Biomaterial maturation of printed cell-free controls with three technical replicates. (A) Amide I region in static cell-free controls with the β-sheet region ranging from 1617 to 1627 cm^–1^ and the random coil (RC) region from 1640 to 1650 cm^–1^ (*n* = 1). (B) Amide I region in HP cell-free controls (*n* = 1). (C) Compressive Young’s modulus of static and HP cell-free controls at 10% strain tested by unconfined compression (*n* = 1). (D) Images of static and HP samples in MSC growth medium.

The compressive Young’s modulus, a measure of hydrogel stiffness, was determined through unconfined compression tests at 10% strain. While the Young’s modulus increased from day 0 to day 14, the application of HP revealed no significant differences in hydrogel stiffness compared to static incubation for 14 and 28 days (Figure 1C). The mean Young’s moduli ranged between 0.029 and 0.125 MPa on days 14 and 28.

Since the increase in β-sheets and stiffness can also be observed by the transparency loss of SF-based hydrogels, pictures were taken on days 0, 14, and 28 (Figure 1D). The hydrogel center remained transparent on day 0, but all gels became opaque by days 14 and 28. Static incubation preserved gel integrity, while stimulation with HP caused slight deformation due to the histology cassettes. Nevertheless, the gels remained largely undamaged.

### 3.2 IFP-MSC Growth Characteristics

The used passages, population doublings within two passages (before seeding into the bioink), and population doubling time of the IFP-MSCs from three donors are given in Table 2. The IFP-MSCs from donor 1 exhibited fewer population doublings and a longer doubling time than those from the other two donors. Nevertheless, the cells from donor 1 were the only ones where the doubling time decreased from 6.4 to 5.4 days within the two passages, whereas the doubling time increased by 1 day for donor 2 cells and 0.4 days for donor 3 cells.

### 3.3 Metabolic Activity Varies between Donors

IFP-MSCs were included in the printed gels to corroborate the biocompatibility of the materials and study the effect of HP stimulation on cell activity. Assessment of metabolic activity demonstrated substantial variability among IFP-MSCs derived from different donors (Figure 2A). The IFP-MSCs from donor 1 were especially active when exposed to HP. While no significant differences were observed, the application of HP resulted in higher metabolic activities compared to static incubation in MSC growth medium or chondrogenic differentiation medium (TGFβ-3-supplemented) on days 14 and 28.

**Figure 2:**
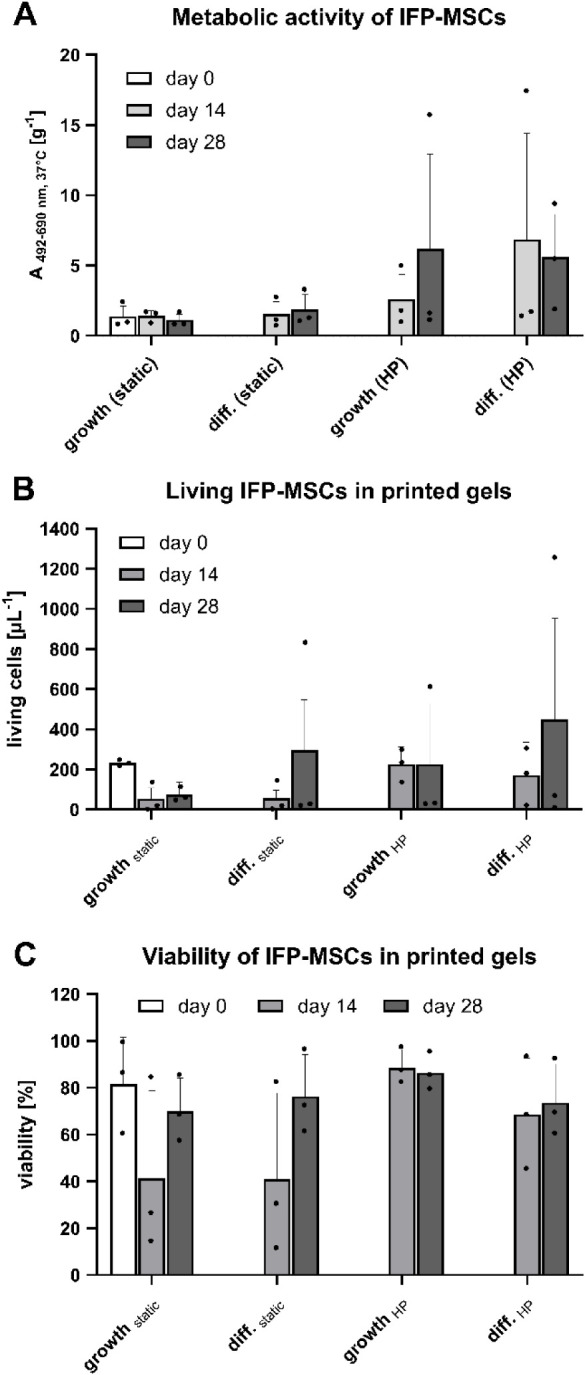
Effect of HP stimulation and application of chondrogenic differentiation medium (TGFβ-3-supplemented) on IFP-MSCs in bioprinted SF10-ECM5-G3 hydrogels on days 0, 14, and 28. Each black dot represents the mean for one biological replicate. (A) Metabolic activity. Data were compared by Kruskal–Wallis tests. No statistical difference was detected (*n* = 3). (B) Living cell number per μL. Data were compared by Kruskal–Wallis tests. No statistical difference was detected (*n* = 3). (C) Cell viability. Data were normally distributed by the Shapiro–Wilk test and compared using two-way ANOVA with Tukey post test. No statistical difference was detected (*n* = 3).

### 3.4 IFP-MSC Viability is Increased by HP

Variability among IFP-MSCs from different donors, previously observed for metabolic activity, was confirmed by confocal microscopy after live and dead staining (Figure 3). The IFP-MSCs from donor 1 led to especially high cell numbers when they were stimulated by chondrogenic differentiation medium (TGFβ-3-supplemented) and/or HP. Conversely, IFP-MSCs from the other two donors yielded low cell numbers in all conditions, with static incubation proving particularly detrimental to cell survival. Interestingly, differences in cell shape could already be observed on day 0, where the IFP-MSCs from donor 1 in Figure 3A adhered to the material, while the IFP-MSCs from donor 2 in Figure 3B remained round. Unexpectedly, the IFP-MSCs from donor 1 exhibited the lowest cell viability (60% ± 13% (SD)) on day 0, compared to the IFP-MSCs from donor 2 (99% ± 1% (SD)) and donor 3 (86% ± 10% (SD)). By day 14, this trend reversed, with the IFP-MSCs from donor 1 exhibiting the highest viability (Figure 2C). In general, mean viabilities between 41% and 89% were obtained, and the application of HP increased cell viability in comparison to static cultivation in MSC growth medium. Nevertheless, no significant differences between static incubation and HP stimulation could be detected. The mean cell viability increased from day 14 to day 28 for all conditions except for growth_HP_ (Figure 2C).

**Figure 3:**
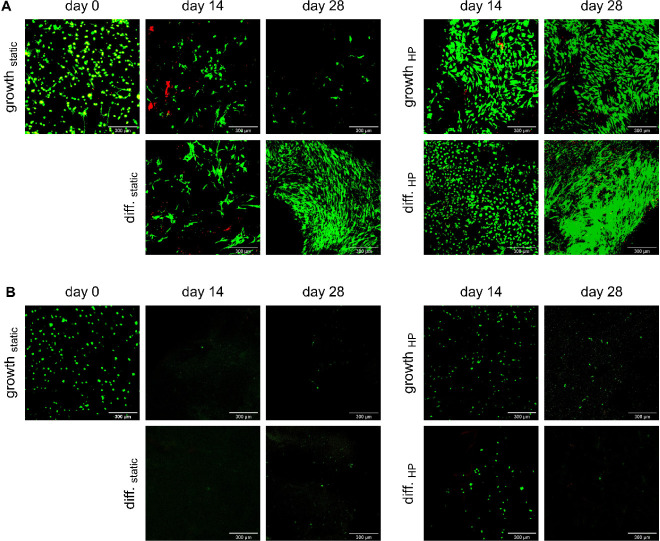
Confocal microscopy images after live and dead staining with 4 μM calcein and 8 μM EthD-1 of IFP-MSCs in bioprinted SF10-ECM5-G3 hydrogels on days 0, 14, and 28. The scale bars indicate 300 μm. (A) Biological replicate with IFP-MSCs from donor 1. (B) Biological replicate with IFP-MSCs from donor 2.

The mean living cell number followed a similar trend of growth_HP_ being the only condition where the living cell number per μL did not increase from day 14 to day 28 (Figure 2B). Growth_HP_ demonstrated the highest mean living cell number on day 14, while diff._HP_ exhibited superior cell numbers on day 28. Nevertheless, no significant differences were observed among the different conditions. Despite the improved proliferation upon HP stimulation within the biological replicates presented in Figure 3A,B, the positive effect could not be statistically confirmed for the means of the biological replicates in Figure 2B. An individual comparison within each biological replicate only revealed a significant cell proliferation increase upon HP stimulation for one of the three biological replicates (data not shown). In addition, no significant differences were observed for the mean living cell numbers per μL when the incubation in MSC growth medium and chondrogenic differentiation medium were compared within each biological replicate (data not shown).

### 3.5 Expression of Chondrogenic Markers is Increased by HP

The differentiation potential of IFP-MSCs in the bioprinted hydrogels was evaluated by gene expression analysis (Figure 4). All Ct values were normalized to *GAPDH* and to the day 0 sample in MSC growth medium by the 2^–ΔΔCt^ method. While *COL10A1* was not expressed in any sample, *COL1A1* and *COL2A1* expression significantly increased from day 14 to day 28 in all conditions except for growth_static_, where it significantly decreased. The expression levels of *COL1A1* and *COL2A1* were markedly elevated in diff._HP_, with mean fold changes of 395 and 78 933, respectively. *ACAN* and *SOX9* were only significantly upregulated from day 14 to day 28 when the samples were incubated in chondrogenic differentiation medium. In contrast, when they were incubated in MSC growth medium, the expression of *ACAN* and *SOX9* significantly decreased from day 14 to day 28. *MMP3* expression was significantly upregulated from day 14 to day 28 in all conditions except for growth_HP_, whereas *MMP13* was significantly downregulated in all conditions except for diff._HP_. Remarkably, growth_HP_ elicited pronounced *MMP3* and *MMP13* expression on day 14.

**Figure 4:**
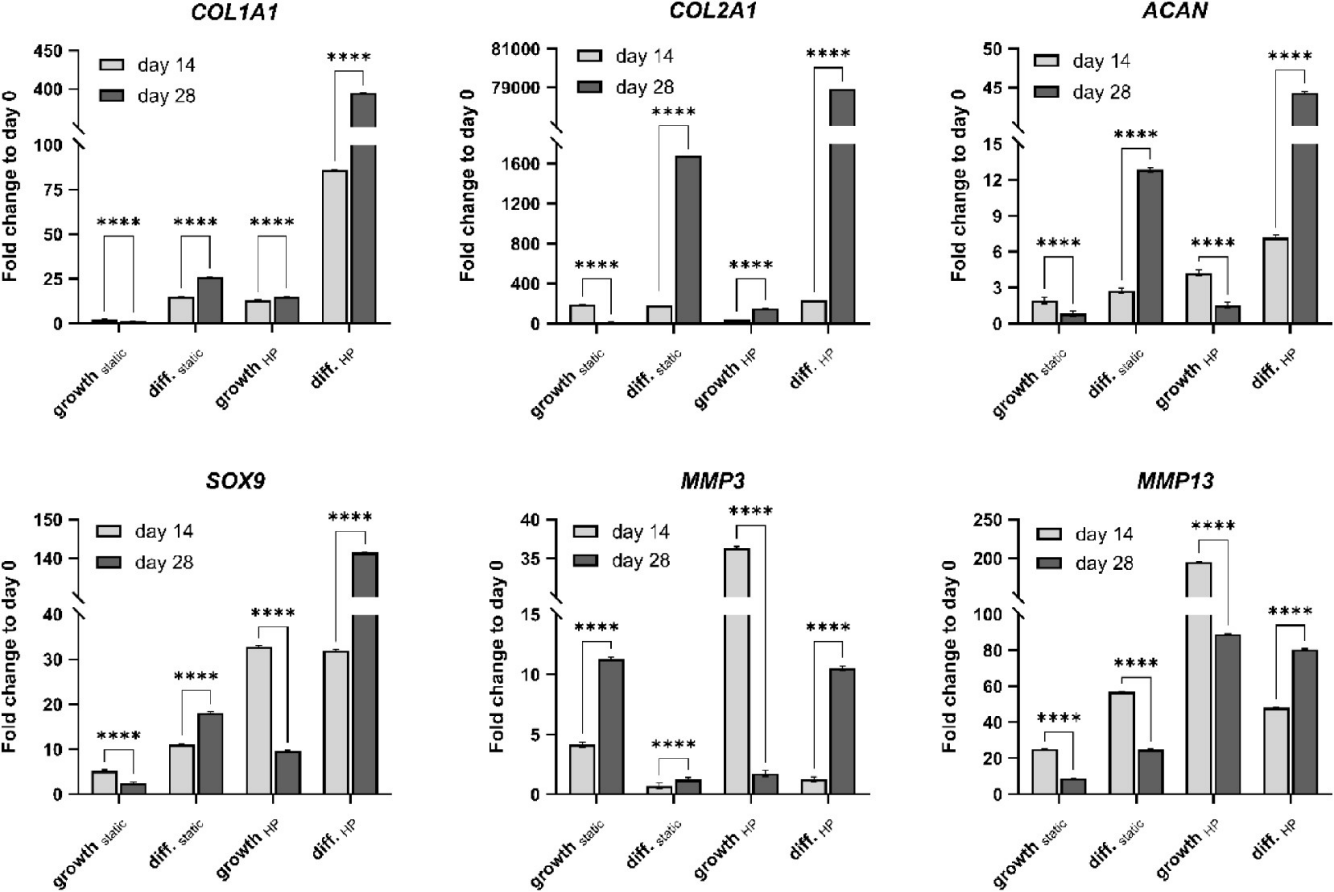
Gene expression analysis of IFP-MSCs in bioprinted SF10-ECM5-G3 hydrogels for *COL1A1*, *COL2A1*, *ACAN*, *SOX9*, *MMP3* and *MMP13*. *COL10A1* was not expressed in any sample. The Ct values were normalized to *GAPDH* and to day 0 samples in the MSC growth medium by the 2^–ΔΔCt^ method. Data was normally distributed (Shapiro–Wilk test) and compared using 2-way ANOVA with Tukey post test. Significance was attributed when *p* < 0.05 (*), *p* < 0.01 (**), *p* < 0.001 (***), and *p* < 0.0001 (****). Only the significant differences between days 14 and 28 were displayed in the bar charts. Further comparisons can be found in Table 3 (*n* = 3).

While only the significant differences between days 14 and 28 were displayed in Figure 4, the effect of HP stimulation and TGFβ-3-supplemented chondrogenic medium application on the expression of chondrogenic markers was further investigated in Table 3. The combination of HP stimulation and chondrogenic differentiation medium (diff._HP_) proved to be the most effective cultivation strategy for chondrogenesis, as it significantly increased the gene expression of *COL1A1*, *COL2A1*, *ACAN,* and *SOX9* in comparison to the other three cultivation conditions on days 14 and 28. Only growth_HP_ led to significantly higher *SOX9* expression on day 14. Investigating whether HP could enhance chondrogenesis alone compared to chondrogenic differentiation medium, growth_HP_ significantly upregulated *ACAN* and *SOX9* expression on day 14 compared to diff._static_. However, diff._static_ proved to be superior in chondrogenesis induction for *COL1A1* and *COL2A1* on day 14 and for all chondrogenic markers on day 28. Nevertheless, HP stimulation significantly upregulated the expression of all chondrogenic markers except for *COL2A1* on day 14, when the samples were incubated in MSC growth medium (growth_HP_ and growth_static_). The same observation was made for diff._static_, which significantly upregulated chondrogenesis in comparison to growth _static_. Although growth _static_ resulted in the lowest expression of chondrogenic markers, with the exception of *COL2A1* on day 14, it still induced upregulation of all markers except for *ACAN* on day 28 compared with the day 0 control. In summary, HP stimulation combined with chondrogenic differentiation medium maximized chondrogenic marker expression, while chondrogenic differentiation medium in static cultivation was more effective than HP alone.

**Table 3:** Effect of HP Stimulation and TGFβ-3-Supplemented Chondrogenic Medium Application on the Expression of Chondrogenic Markers^a^

**day 14**		***COL1A1***	***COL2A1***	***ACAN***	***SOX9***
diff._HP_	growth_HP_	**+**	**+**	**+**	**-**
	diff._static_	**+**	**+**	**+**	**+**
	growth_static_	**+**	**+**	**+**	**+**
growth_HP_	diff._static_	**-**	**-**	**+**	**+**
	growth_static_	**+**	**-**	**+**	**+**
diff._static_	growth_static_	**+**	**-**	**+**	**+**

**day 28**		***COL1A1***	***COL2A1***	***ACAN***	***SOX9***
diff._HP_	growth_HP_	**+**	**+**	**+**	**+**
	diff._static_	**+**	**+**	**+**	**+**
	growth_static_	**+**	**+**	**+**	**+**
growth_HP_	diff._static_	**-**	**-**	**-**	**-**
	growth_static_	**+**	**+**	**+**	**+**
diff._static_	growth _static_	**+**	**+**	**+**	**+**

a Upregulation in the condition in the first subcolumn of day 14 or day 28 in comparison to the second subcolumn was Indicated with + and Downregulation with -. All differences (+ and -) were statistically significant (****) (*n* = 3).

### 3.6 Staining for sGAGs and Collagen is Partially Increased by HP

The hydrogel composition and cell distribution were analyzed by staining 10 μm thin histological sections of hydrogels with IFP-MSCs from donor 1 and comparing them to those of an OA human meniscus. Hematoxylin–Eosin staining demonstrated uniformly low cellular density across all experimental conditions (Figure 5). The meniscus exhibited a slightly higher cellular density. The low cell density was also observed through Alcian Blue and Nuclear Fast Red staining (Figure 6). The hydrogel displayed a predominantly pink coloration on day 0, which persisted in growth_static_ on day 14. In contrast, the gels in the other experimental groups exhibited more violet coloration, indicating sGAG deposition. The combination of HP and TGFβ-3-supplemented chondrogenic differentiation medium even facilitated more intense sGAG formation on day 28, as revealed by the blue rings around some cells. Despite the observed sGAG deposition, the levels were still insufficient to replicate the intense blue staining characteristic of the meniscus.

**Figure 5:**
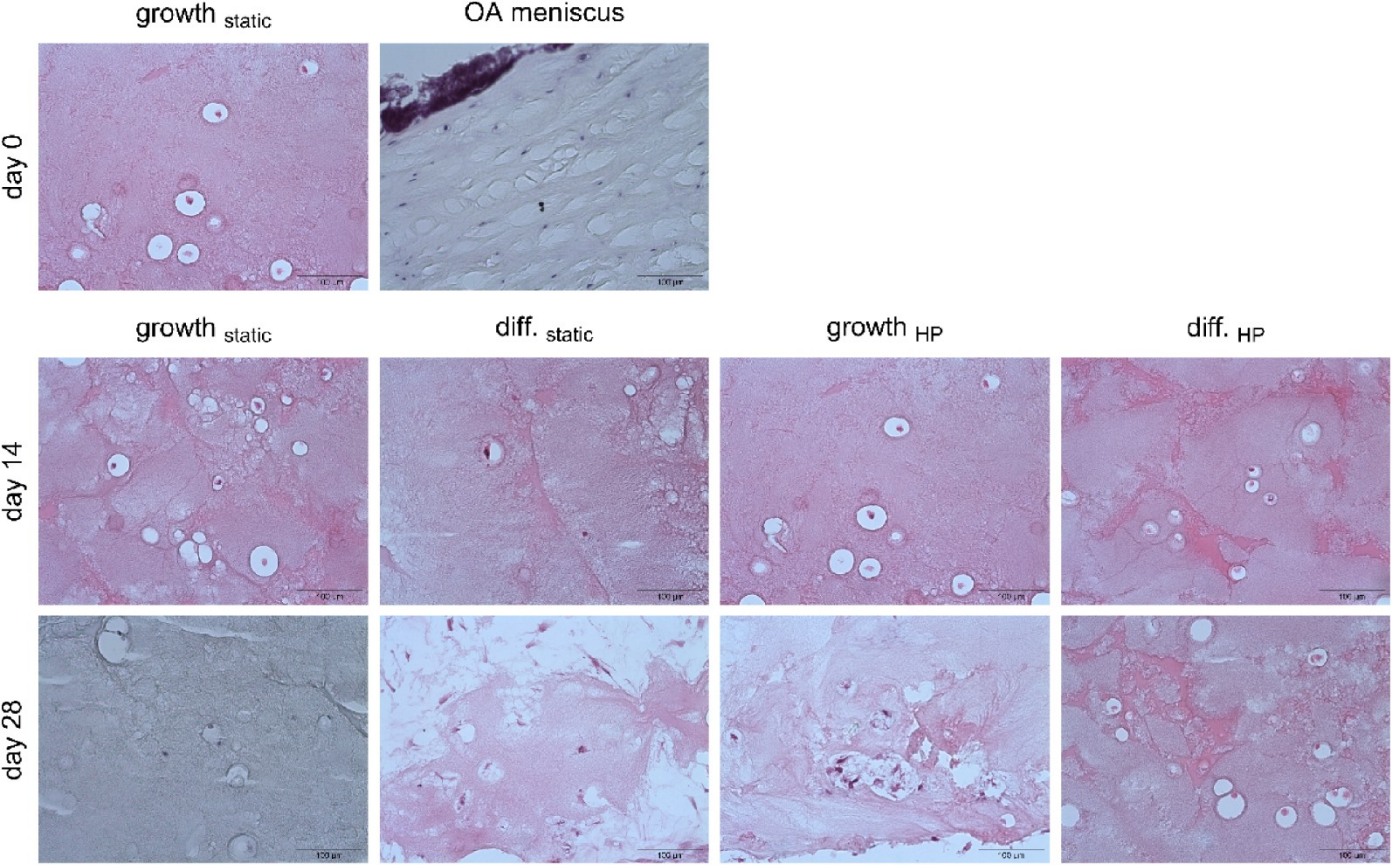
Hematoxylin–Eosin staining of 10 μm thin sections of hydrogels with IFP-MSCs from donor 1. IFP-MSC nuclei are colored dark red to violet, while the background is colored pink. Images were captured at 20× magnification, and the scale bars indicate 100 μm (*n* = 1).

**Figure 6:**
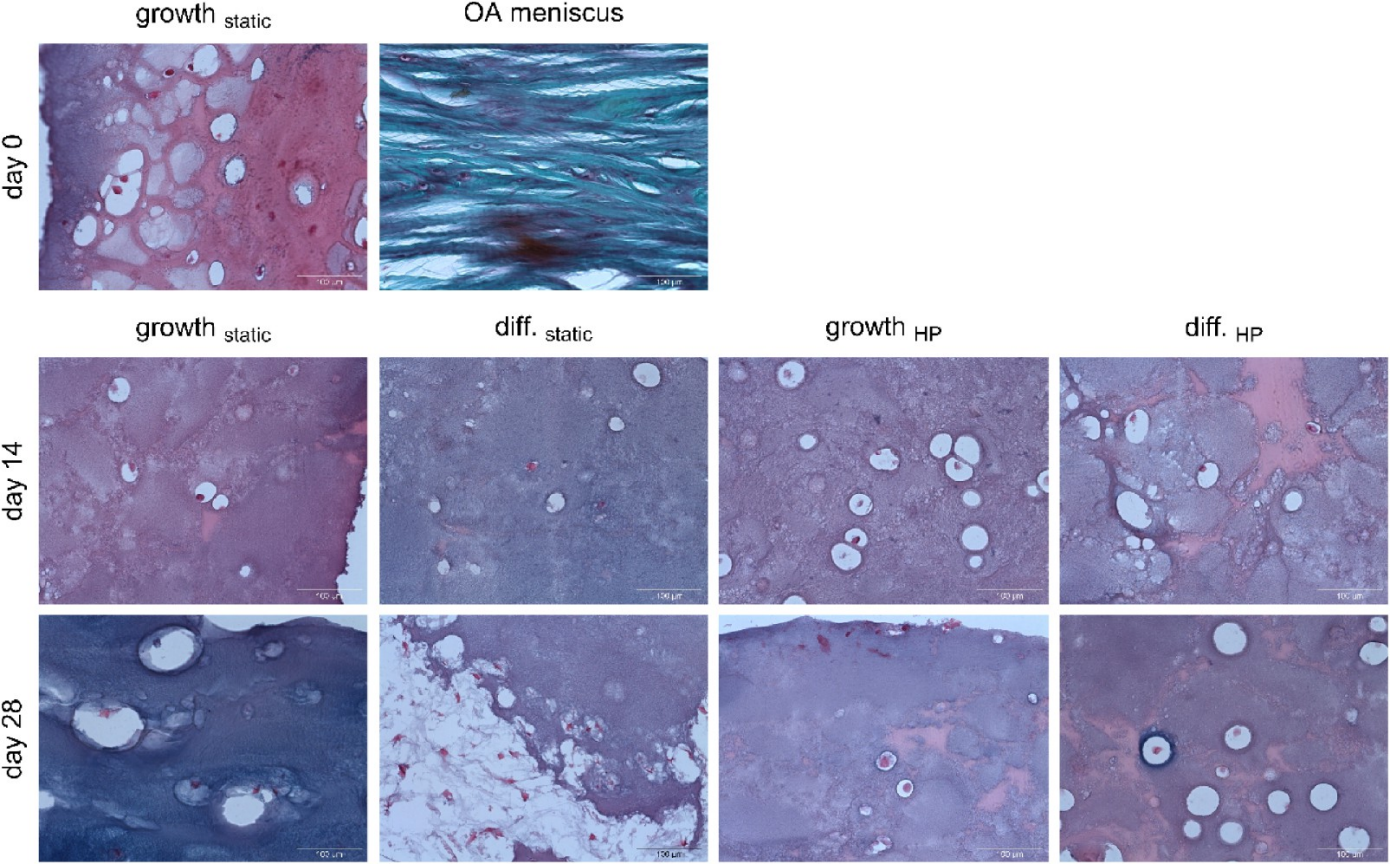
Alcian Blue and Nuclear Fast Red stainings of 10 μm thin sections of hydrogels with IFP-MSCs from donor 1. IFP-MSC nuclei are colored in red, while sGAGs are colored in blue. Images were captured at 20× magnification, and the scale bars indicate 100 μm (*n* = 1).

Azan staining was employed to investigate the presence of collagen (Figure 7). The hydrogels exhibited a light blue coloration in the center, transitioning to a darker blue at the edges. Furthermore, the combination of HP and chondrogenic differentiation medium resulted in the emergence of dark blue spots on days 14 and 28, indicative of increased collagen deposition. Nevertheless, no cells could be detected within the core of these dark blue regions anymore. Despite the increased collagen deposition, the hydrogels failed to reproduce the intense blue staining of the collagen fibers in the OA meniscus. Alcian Blue staining (Figure 6) and Azan staining (Figure 7) also illustrated that the hydrogels lacked structural organization in comparison to the meniscus, where the collagen fibers were aligned in parallel.

**Figure 7:**
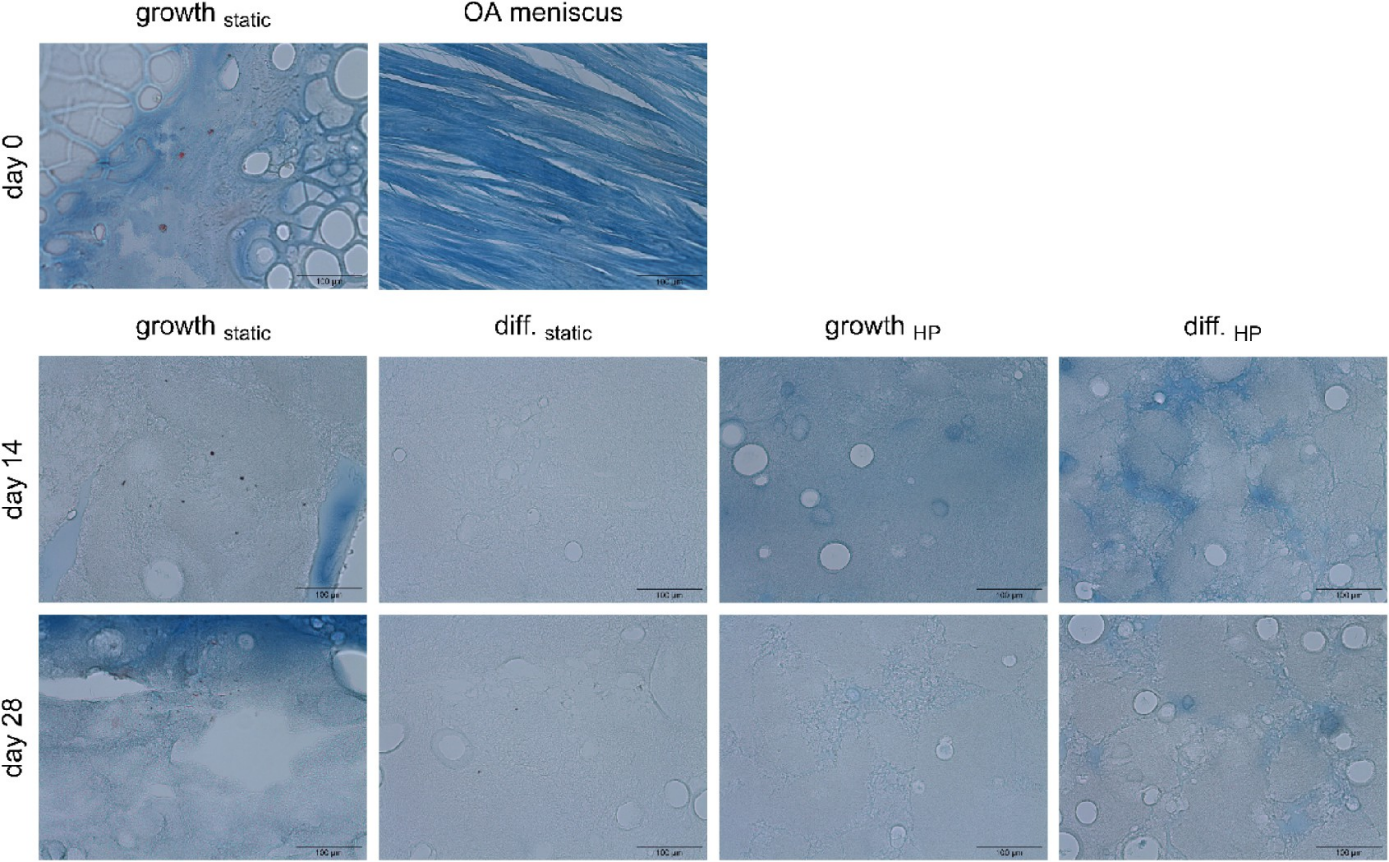
Azan staining of 10 μm thin sections of hydrogels with IFP-MSCs from donor 1. IFP-MSC nuclei are colored red, while collagen is colored in blue. Images were captured at 20× magnification, and the scale bars indicate 100 μm (*n* = 1).

## 4 Discussion

This study investigated the effects of HP stimulation on 3D-printed cell-free and 3D-bioprinted cell-laden SF10-ECM5-G3 hydrogels. Since HP stimulation is known to promote β-sheet formation and stiffening of SF polymer scaffolds, ATR-FTIR spectroscopy and unconfined compression were applied to cell-free controls for mechanical property evaluation.^35^ However, stimulation with up to 10 MPa at 0.7 Hz did not significantly alter the secondary SF structure or the compressive Young’s modulus (Figure 1). According to the study by Yazawa et al., stimulation of dried SF films with 0–75 MPa HP for 5 min did not induce β-sheet formation, while 100–980 MPa HP did and also increased the Young’s modulus in tensile tests.^35^ These findings confirm that the application of HP as low as 10 MPa could preserve the secondary structure and Young’s modulus progression similar to static incubation. Also, Gunja et al. demonstrated that HP stimulation of poly-L-lactic acid scaffolds did not directly affect the mechanical properties of cell-free controls.^25^ Only when rabbit meniscus cells were seeded on the scaffolds and stimulated with 10 MPa HP to produce more collagen and sGAGs were the compressive properties significantly increased.^25^ This finding suggests that the compressive Young’s modulus after HP stimulation (0.054–0.125 MPa on day 28 in Figure 1) would likely be even higher for the bioprinted samples with IFP-MSCs in our study and may surpass the values observed in human menisci (83.4 kPa).^36^

3D bioprinting of IFP-MSCs in the hydrogels resulted in substantial variability in metabolic activity and cell number (Figure 2A,B). The differences were particularly evident after HP stimulation, which promoted metabolic activity and proliferation, assessed by cell number increase, in the IFP-MSCs from donor 1 more than in the others. The observed differences are likely attributable to variations in age and disease (Table 1). While donor 1 had ACL and meniscus tears, donors 2 and 3 suffered from degenerative diseases, whose highly inflammatory environment might have impacted the IFP-MSCs more than ACL and meniscus tears. Inflammation in OA can have different impacts on the proliferative and chondrogenic potential of IFP-MSCs. On the one hand, it has been demonstrated that IFP-MSCs from OA patients retain their chondrogenic potential and do not necessarily display constitutive inflammatory activation.^37^ For example, Liu et al. reported that IFP-MSCs from OA donors exhibited no significant differences in their chondrogenic capacity and DNA content after proliferation in comparison to IFP-MSCs isolated from patients undergoing ligament reconstruction.^38^ On the other hand, Bravo et al. hypothesized that IFP-MSCs from OA patients are altered by OA inflammation, which was confirmed by a significant decrease in the expression of cartilage factors TGFβ and FGF2 and the anabolic factor MMP3.^39^ Inflammation might prime IFP-MSCs not only in OA but also during ACL and meniscus injuries. Rai et al. compared cytokine and growth factor concentrations in the synovial fluid after ACL tears and during OA.^40^ They found that ACL tear patients had higher levels of four anti-inflammatory molecules (interleukin-4 (IL-4), IL-5, placenta growth factor 1, and IL-13) and two pro-inflammatory molecules (tumor necrosis factor-α (TNF-α) and beta nerve growth factor).^40^ Despite their role as key inflammatory mediators in OA pathogenesis, IL-6 and IL-1β levels did not differ significantly between the two groups.^40,41^ However, Stocco et al. demonstrated the absence of lymphocytic infiltration, which is present in OA patients, in IFP biopsies of ACL tear patients after the acute inflammation phase.^42,43^ This finding suggests that ACL injury-associated inflammation has a weaker effect on the IFP than OA-associated inflammation. Furthermore, Kouroupis et al. observed diminished proliferation when IFP-MSCs from patients with ACL injury were primed with OA-related cytokines.^44^

Besides various diseases, different ages (22, 42, and 82 years, as shown in Table 1) could have influenced cell proliferation and metabolic activity. The IFP-MSCs from the youngest donor demonstrated remarkable proliferation and activity in response to HP stimulation, while the IFP-MSCs from the other donors yielded low cell numbers and activity measures under all conditions (Figure 2A,B). The proliferation and differentiation potential of subcutaneous adipose tissue-derived MSCs (AMSCs) are known to be negatively affected by donor age.^45^ In contrast, Garcia et al. did not report significant variability in the population doubling time of IFP-MSCs from six donors, aged 35–79 years, who underwent autologous chondrocyte implantation or TKA.^46^ Their mean population doubling time of approximately 6.5 days after passage 1 is slightly higher than the 3.6–6.4 days observed in our study.^46^ Other studies report even shorter population doubling times for OA IFP-MSCs.^46–48^ Tanimoto et al. obtained IFP-MSCs from OA patients, aged 70.6 ± 8.1 years, and reported doubling times of 40.5 ± 1.8 h.^48^ Stocco et al. obtained IFP-MSCs from OA patients, aged 64–79 years, and reported doubling times of 42.7 ± 3.8 h.^47^ Their cells did not reach senescence within 20 passages.^47^ The low population doubling times reported in the literature might suggest that age and disease do not necessarily affect IFP-MSC proliferation in 2D culture. IFP-MSCs from more donors would have been desirable in our study to identify whether donor age, disease, or both correlate with metabolic activity and/or proliferative capacity. Another cause of variability could be the different passage numbers seeded into the bioink. The high passage numbers of the cells from donors 2 and 3 (passages 6 and 7, as shown in Table 2) could have reduced their proliferation potential. Radhakrishnan et al. previously demonstrated that human IFP-MSCs only maintained their self-renewability and ability to differentiate into the three germ layer cell types until passage 4.^49^ Culturing the cells beyond passage 6 in 2D culture led to a decline in the proliferative marker nucleostemin, a longer population doubling time, and a shift toward neuronal differentiation.^49^ These findings contrast with the previously described studies by Garcia et al. and Stocco et al.^46,47^ In our study, the population doubling time of IFP-MSCs from donors 2 and 3 increased before they were seeded into the bioink, which could have already indicated a low proliferation potential. Nevertheless, more passages are needed to confirm the proliferation potential. The chondrogenic differentiation potential was not affected compared to that of the cells from donor 1. Besides the IFP-MSC characteristics, the use of freshly prepared SF solution, ECM powder, and a new batch of HRP may also have contributed to the observed variations. The differences in cell shape observed on day 0 in Figure 3 suggested that the adhesion ability of the IFP-MSCs from donors 2 and 3 was impaired or that the available binding sites of ECM and G were compromised.

Cell viability also displayed variability, as static incubation proved to be detrimental to the survival of IFP-MSCs from donors 2 and 3 (Figure 2C). In comparison, HP stimulation increased cell viability in MSC growth medium, which is in accordance with the results of Li et al.^50^ They reported that rabbit IFP-MSCs exhibited improved viability and proliferation in alginate beads when exposed to 5 MPa HP.^50^ Our study resulted in mean cell viabilities between 60% and 99% on day 0, which showed that the majority of cells survived the printing and cross-linking process (Figure 2C). During further cultivation, cell viability and proliferation could have been affected by the increasing hydrogel stiffness (Figure 1C). Hydrogels with high polymer concentrations have already been shown to restrict nutrient transport due to decreased permeability.^51^ Specifically, SF hydrogels with abundant β-sheet structures hinder the permeation of nutrients into the hydrogels.^52^ For instance, Wang et al. attributed the decreased proliferation of bone marrow-derived MSCs (BMSCs) in 12% (w/v) SF hydrogels compared to 4% (w/v) SF hydrogels to mass transport limitations and mechanical restrictions.^53^ Nevertheless, Zhao et al. reported that HP could reverse the negative influence of dense hydrogels on cell viability and proliferation by improving nutrient transport through increased permeability.^54^ The results of Zhao et al. suggest that improved nutrient transport may have contributed to the increased cell viability observed after HP stimulation in comparison to static incubation in MSC growth medium in our study (Figure 2C).

While HP stimulation only partially affected cell viability and proliferation, the influence of TGFβ-3-supplemented or TGFβ-free HP stimulation on chondrogenic marker expression was more pronounced. The combination of HP and chondrogenic differentiation medium (TGFβ-3-supplemented) significantly elevated the expression levels of *COL1A1*, *COL2A1*, *ACAN,* and *SOX9* on day 28 in comparison to the other conditions (Figure 4 and Table 3). These results were in accordance with literature findings regarding the exposure of hydrogel-embedded human MSCs to HP and chondrogenic differentiation medium.^21–24,38^ For example, Ogawa et al. found that adipose-derived MSCs in collagen hydrogels demonstrated up to 12-fold increased expression of *COL2A1*, *ACAN,* and *SOX9* upon stimulation with cyclic HP up to 0.5 MPa, normalized to the day 0 control.^23^ Our study resulted in up to 395-fold increases for *COL1A1*, 45-fold for *ACAN*, and 142-fold for *SOX9* in normalization to the day 0 control (Figure 4). *COL2A1* exhibited especially high fold increases in expression, up to 78933, as it was barely expressed in the day 0 controls. The investigation into whether TGFβ-free HP stimulation could enhance chondrogenesis showed that static incubation with a chondrogenic differentiation medium proved to be superior in the expression of all chondrogenic markers on day 28. The initial promotion of TGFβ-free HP stimulation of *ACAN* and *SOX9* expression was not only demonstrated in our study but also by Puetzer et al.^55^ They reported that cyclic HP of 7.5 MPa initially upregulated *ACAN* and *SOX9* expression in human adipose-derived MSCs embedded in agarose constructs without TGFβ.^55^ However, *COL2A1* was only expressed in the HP-stimulated samples on day 14, and the expression of *ACAN* and *SOX9* decreased below the expression levels of the unloaded control on day 14.^55^

The superiority of incubation with TGFβ was also demonstrated by Kisiday et al., who used uniaxial unconfined compression to introduce HP in agarose hydrogels seeded with equine BMSCs.^56^ While HP significantly increased proteoglycan synthesis in the absence of TGFβ, the addition of TGFβ-1 to unloaded controls significantly increased proteoglycan synthesis in comparison to loaded samples without TGFβ.^56^ Despite the minimized effect of TGFβ-free HP stimulation on chondrogenesis induction in comparison to incubation with TGFβ-3, TGFβ-free HP stimulation was still able to significantly upregulate the expression of all chondrogenic markers on day 28 in comparison to static incubation in MSC growth medium (Figure 4 and Table 3). A similar observation was made by Miyanishi et al., who reported that the application of 10 MPa HP significantly increased the expression of *COL2A1*, *ACAN,* and *SOX9* in TGFβ-free human MSC pellet cultures.^57^ While the unloaded control with TGFβ-3 resulted in significantly higher expression levels than the HP-loaded samples without TGFβ, the combination of HP and TGFβ-3 further increased the expression levels.^57^ In summary, our study demonstrated that TGFβ-3-supplemented HP stimulation led to the highest expression levels of chondrogenic markers, followed by TGFβ-3-supplemented static incubation and then TGFβ-free HP stimulation.

*MMP13* and *COL10A1* are commonly used hypertrophy markers.^58^ Despite the established tendency of MSCs to exhibit hypertrophy during chondrogenic differentiation, *COL10A1* expression was not detected in any of our samples.^59^ This finding could align with literature indicating IFP-MSCs’ limited propensity to undergo endochondral ossification.^60,61^ Vinardell et al. differentiated porcine BMSCs, IFP-MSCs, and synovial membrane-derived stem cells (SMSCs) in agarose hydrogels in chondrogenic medium (with TGF-β3) and evaluated type 1, 2, and 10 collagen content with immunohistochemistry.^60^ While BMSCs followed an endochondral pathway with increased collagen type X expression, IFP-MSCs and SMSCs increased collagen type 1 expression, reduced sGAG production, and were suspected to undergo fibrous dedifferentiation.^60^ Similarly, Lopa et al. compared IFP-MSCs to subcutaneous AMSCs from 25 OA patients undergoing TKA.^61^ IFP-MSCs demonstrated significantly higher chondrogenic potential and significantly lower *COL10A1* expression than AMSCs.^61^ In both cell types, *COL1A1* expression was 50-fold greater than *COL10A1* expression.^61^ *COL10A1* expression during hypertrophy is usually accompanied by *MMP13* expression.^58^ However, *MMP13* expression is not necessarily associated with hypertrophy and *COL10A1* expression. For example, *MMP13* expression in human chondrocytes from OA donors was elevated in comparison to donors without joint disease, while *COL10A1* expression was not upregulated.^62^ These results indicated a decoupling of *MMP13* and *COL10A1* expression and that *MMP13* expression may be upregulated through hypertrophy-independent mechanisms, such as inflammation and mechanical stress.^58^ Furthermore, Ronzière et al. compared *MMP13* and *COL10A1* expression during chondrogenic differentiation of human BMSCs and AMSCs with TGF-β3 and/or bone morphogenetic protein-2.^63^ BMSCs expressed *MMP13* in all conditions from day 1 onward, whereas *COL10A1* expression started on day 12. AMSCs also expressed *MMP13* from day 1 onward, but *COL10A1* expression was only detected on day 24 with chondrogenic differentiation medium.^63^ The universal expression of *MMP13* in our study, in the absence of *COL10A1* expression, supports the hypothesis of *MMP13* expression via alternative pathways other than hypertrophy.^58,62^

The increased deposition of sGAGs and collagen after TGFβ-3-supplemented HP stimulation could also be observed through Alcian Blue and Azan staining, as shown in Figures 6 and 7. Hydrogels primarily exhibited uniform violet or light blue staining, with darker blue rings around certain cells, suggesting localized ECM deposition. The visibility of differences in ECM deposition in hydrogels by histological staining has already been described as limited in other studies. For example, Liu et al. reported that TGFβ-3-supplemented HP stimulation only led to slightly more intense staining for sGAGs and collagen in 2% (w/v) agarose gels with IFP-MSCs.^64^ In contrast, Gunja et al. reported that TGFβ-1 application to poly-L-lactic acid scaffolds with rabbit meniscus cells clearly increased the density of sGAGs and collagen in the periphery of the hydrogels.^25^ The application of 10 MPa at 0 Hz further promoted sGAG and collagen staining intensity, as observed with safranin O/fast green and picrosirius red staining.^25^ A favored ECM deposition in the peripheral region was also observed by Carroll et al.,^65^ who exposed porcine IFP-MSCs in agarose hydrogels to 10 MPa HP and found the predominant localization of Alcian Blue staining for sGAGs to be in the peripheral region, with minimal variation between HP-treated and unloaded groups.^65^ In our study, increased ECM deposition at the edge was observed only for some statically incubated hydrogels (Figures 6 and 7), suggesting that HP application improved nutrient and gas distribution, leading to more uniform ECM deposition.

The staining of the histological hydrogel sections also highlighted the differences with the meniscus sections and, therefore, the limitations of our study. On the one hand, the hydrogels exhibited lower cell numbers than the meniscus (Figure 5), although the OA meniscus tissue might already have been hypocellular. On the other hand, the uniform hydrogel conformation failed to recapitulate the fibrous architecture of the meniscus collagen fibers, as shown in Figures 6 and 7. While the collagen fibrils in the meniscus are aligned in fibril bundles with a diameter of 5–10 μm and then in fiber bundles with a diameter of 10–150 μm, visible in Figures 6 and 7, the collagen fragments introduced by the addition of decellularized meniscus ECM exhibit much smaller diameters.^66^ For example, Gong et al. reported increasing collagen fiber thickness between 75.5 and 101.1 nm for increasing hydrogel concentrations of decellularized cartilage ECM (1–10 mg/mL) from porcine ears.^67^ Therefore, it is not surprising that neither the added collagen fragments nor newly produced collagen from the limited cell population resulted in a noticeable fibrous structure at this stage. However, Costa et al. were able to detect collagen fiber alignment after co-printing a gellan gum/fibrinogen bioink with porcine meniscus cells and an SF methacrylate ink for fibrocartilaginous regeneration.^11^ Although images of Safranin–O, Masson’s trichrome, and Alcian Blue/Sirius Red staining did not indicate specific collagen fiber alignment after subcutaneous implantation in mice, it was detected in higher-resolution images by polarization of picrosirius red-stained collagen fibers.^11^ Their study highlighted the ability of the 3D-printed grid structure to promote the alignment of collagen fibers, replicating the structural organization found in the native meniscus, and suggested that the picrosirius red polarization detection method might have revealed collagen fiber alignment in our study.

Nevertheless, we suspect that higher cell viability and proliferation need to be achieved to increase collagen and sGAG deposition for fibrous structure formation in our hydrogels. Therefore, the effects of shear stress during 3D bioprinting with different needle sizes and the effects of cross-linking by HRP and H_2_O_2_ on the initial cell viability should be examined for the IFP-MSCs of each donor separately in the future. IFP-MSCs from more donors would be required to identify whether donor age, disease, or both correlate with metabolic activity and/or proliferative capacity. Since our 3D bioprinted samples contained only a grid infill, 3D bioprinting the hydrogel in accordance with the natural architecture of the meniscus might enable aligned collagen deposition in future studies and increase the performance of the construct for meniscus regeneration.

## 5 Conclusion

This study used 3D bioprinted SF-ECM-G hydrogels with human IFP-MSCs for meniscus tissue engineering. The samples were cross-linked with HRP and H_2_O_2_ and subjected to cyclic HP stimulation of 10 MPa to mimic mechanical loading in the knee joint. While HP was found to have no effect on the secondary SF structure and the compressive Young’s modulus of cell-free controls, its effect on cell proliferation was demonstrated only in the cells of one donor. A more pronounced response in the cells of all donors was observed by gene expression analysis. Specifically, TGFβ-3-stimulated HP application increased the expression of chondrogenic markers and facilitated sGAG and collagen deposition. Our findings emphasize the significance of applying physiological mechanical stimuli to SF-based 3D bioprinted hydrogels for the development of implants aimed at meniscus regeneration.

## Data Availability

The data that support the findings of this study are available from the corresponding author upon reasonable request.
